# Using primary care databases for addiction research: An introduction and overview of strengths and weaknesses

**DOI:** 10.1016/j.abrep.2022.100407

**Published:** 2022-01-13

**Authors:** Daniel Kotz, Amy O'Donnell, Sterling McPherson, Kyla H. Thomas

**Affiliations:** a Institute of General Practice, Addiction Research and Clinical Epidemiology Unit, Medical Faculty of the Heinrich‐Heine‐University Düsseldorf, Germany; b Research Department of Behavioural Science and Health, Institute of Epidemiology and Health Care, University College London, UK; c Usher Institute of Population Health Sciences and Informatics, University of Edinburgh, UK; d Population Health Sciences Institute, Newcastle University, Newcastle upon Tyne, UK; e Program of Excellence in Addictions Research and the Analytics and PsychoPharmacology Laboratory (APPL), Washington State University, Elson S. Floyd College of Medicine, USA; f Population Health Sciences, Bristol Medical School, UK

**Keywords:** Addiction research, Primary care database, Retrospective cohort studies

## Abstract

Primary care databases extract and combine routine data from the electronic patient records of various participating practices on a regular basis. These databases can be used for innovative and relevant addiction research, but such use requires a thorough understanding of how data were originally collected and how they need to be processed and statistically analysed to produce sound scientific evidence. The aims of this paper are therefore to (1) make a case for why primary care databases should be considered more frequently for addiction research; (2) provide an overview of how primary care databases are constructed; (3) highlight important methodological and statistical strengths and weaknesses of using primary care databases for research; and (4) give practical advice about how a researcher can get access to databases. Three major primary care databases from the UK serve as examples: Clinical Practice Research Datalink (CPRD), The Health Improvement Network (THIN), and QResearch.

## Highlights

• Primary care databases can be used for innovative and relevant addiction research.
• Strengths include ready access of real-world longitudinal patient data for research.
• Limitations include missing data and lack of accuracy and completeness of data.

## 1 Background and rationale for using primary care data for addiction research

The use of data routinely collected by primary care professionals (in particular general practitioners, GPs) for research purposes has several potential advantages, including access to a general patient population with all kinds of diseases - including addictive behaviours - and long-term longitudinal routine data collection. These databases can be more widely used to address innovative and relevant questions in addiction research.

The aims of this paper are therefore to (1) make a case for why primary care databases should be considered more frequently for addiction research; (2) provide an overview of how primary care databases are constructed; (3) highlight important methodological and statistical strengths and weaknesses of using primary care databases for research; and (4) give practical advice about how a researcher can get access to databases in the UK. In particular, we stress that as the main function of electronic data capture in primary care is to inform day-to-day clinical practice (Gregory, 2009), their use for research purposes requires a thorough understanding of how data were originally collected and how they need to be processed and statistically analysed to produce sound scientific evidence (de Lusignan and van Weel, 2006, Herrett et al., 2015, Lawrenson et al., 1999).

Although primary care research databases are available in various countries, we focus here on the UK as an exemplar case. In comparison to other developed countries, the UK was a relative ‘early adopter’ of electronic patient records in primary care, meaning there is now over 30 years’ of data held in GP information systems (Vezyridis and Timmons, 2016, Williams et al., 2012). Additionally, there is a well-established academic community focused on research using UK primary care data, providing us with several relevant examples in the addictions field on which we can draw.

## 2 Introducing UK primary care data

UK primary care has essentially been fully computerised since the late 1990s, with GPs instructed to ‘add at least one clinical code’ per consultation to a patient’s electronic record (Department of Health, Royal College of General Practitioners, & British Medical Association, 2011). Additionally, the introduction of the UK Quality and Outcomes Framework (a pay-for-performance scheme for GPs) has meant that general practices are required to record detailed, standardised information on a range of specific priority medical conditions to qualify for payment (Gnani & Majeed, 2006).

The major primary care clinical computer systems currently used in the UK include EMIS (Egton Medical Information System), SystmOne, and Vision (Williams et al., 2012). UK primary care computer systems record data in two ways. First, via date-stamped coded (or structured) data, where the data entrant selects the most appropriate clinical term using a keyword search or standardised template to represent the main purpose of the consultation event (whether this refers to a presenting complaint, a diagnosis, procedure or administrative term), with additional clinical terms added as necessary. Second, most systems also allow the entry of free text or narrative as part of the record of the patient encounter. Narrative free text may be used to qualify any clinical term, and thus place the coded information within the overarching context of the patient’s ‘story’, including non-systematically collected behaviours (Department of Health et al., 2011).

Until recently, UK primary care predominately used Read codes for the purposes of recording structured clinical data. Read codes are a hierarchically-arranged controlled standard clinical vocabulary (Robinson, Schulz, Brown, & Price, 1997) which support detailed encoding of multiple patient phenomena, including: demographic details; clinical signs; symptoms and observations; laboratory tests and results; and diagnoses. Two different Read code versions exist: READ version 2 (commonly known as 5-Byte READ due to its five character code structure) and READ version 3 (Clinical Terms Version 3; CTV3). However, in addition to the inconsistencies arising from the continued use of these two different versions in UK primary care, other drawbacks of the Read code system include: incorrect/outdated content; lack of capacity to accommodate new content; poor specificity; and limited interoperability with other clinical data systems (Department of Health et al., 2011, NHS Digital, 2016).

To address these limitations, and to ensure greater consistency in clinical data across care settings, the UK National Health Service has been rolling out a new coding system across primary care since 1st April 2018: SNOMED CT (Systematised Nomenclature for Medicine—Clinical Terms) (UK Terminology Centre, 2011). SNOMED CT provides a comprehensive, multilingual clinical healthcare terminology that is mapped to other international standards and is currently used in over eighty countries (SNOMED International, 2020). Compared to either Read code version, SNOMED CT permits increased levels of detail, accuracy, and hierarchical complexity. Crucially, and again unlike the Read code system, SNOMED CT allows the addition of new descriptions to a concept while retaining outdated descriptions. While clinical staff can select the term they wish to record, the system correctly recognises when different terms have the same conceptual meaning (NHS Digital, 2016).

## 3 Introducing UK primary care databases

We now focus on three exemplar databases from the UK which have been most frequently used for both national and international research purposes (Vezyridis & Timmons, 2016). First, the Clinical Practice Research Datalink (CPRD) is the largest primary care research database in the UK (Herrett et al., 2015). CPRD currently collects patient electronic health records from GP practices which use either the Vision or EMIS patient management software systems. Data are therefore provided as separate datasets: CPRD GOLD (Vision system) (Herrett et al., 2015) and the larger CPRD Aurum (EMIS system) (Wolf et al., 2019). In 2020, the two datasets combined contained 14.9 million current patients (22.5% of the UK population) from 1,642 practices (18.3% of the UK practices; https://www.cprd.com/primary-care). CPRD data can be linked to other data sources such as death registrations (Office for National Statistics Mortality Database), hospitalisations (Hospital Episode Statistics), cancer registrations (National Cancer Registration and Analysis Service) and mental health (Mental Health Dataset) (Padmanabhan et al., 2019). Data may also be linked to measures of relative deprivation for general practices and individual patients (Padmanabhan et al., 2019).

Second, The Health Improvement Network (THIN) database uses data extracted from Vision primary care computer software dating back to 1994. THIN currently contains the electronic health records of 19.7 million patients from over 850 general practices (2.9 million active patients), and is broadly representative in terms of age, sex, deprivation, geographical distribution, and common long term conditions (Blak et al., 2011, THIN (The Health Improvement Network). , 2020). The database contains details of symptoms, diagnoses, prescriptions, test results, health indicators and the Townsend deprivation index (a composite measure of social deprivation presented as quintiles) (Townsend, Phillimore, & Beattie, 1988). Clinical data in THIN are catalogued using Read codes and SNOMED CT Codes in England, ICD-10 codes, and drug Anatomical Therapeutic Chemical (ATC) codes which identify prescribed medications. The patient is identified only by a code allocated by the primary care practice system and cannot be identified outside the practice. Primary care practitioners contributing data to THIN receive training to ensure consistent recording of important clinical outcomes and indicators, including mental health and smoking status.

Third, the QResearch database (www.qresearch.org) was established in 2003 and is a not-for-profit venture currently supported by the University of Oxford and EMIS Health. The data come from approximately 1,500 general practices throughout the UK using the EMIS clinical computer system. At current, the database holds anonymised health records of over 35 million patients who are currently registered with the practices as well as historical patients who may have died or left. The database has been linked to cause of death data, cancer and hospital data at individual patient level with linkages extending back as far as 1993.

## 4 Getting access to UK databases

Preparatory work is needed before accessing a primary care database (see also Box 1). To access CPRD data for research purposes, the researchers’ institution first needs to be eligible, followed by the submission of a protocol for approval by CPRD’s Independent Scientific Advisory Committee (see: https://www.cprd.com/Data-access). Further information including the protocol application form are available from https://cprd.com/research-applications. Prices exclude VAT and range from €17,000 (individual study licence) to €84,000 (multi-study licence) for non-commercial studies using primary care data only. More information on pricing is available from https://cprd.com/pricing.

Box 1 **Ten basic steps in conductinga primary care database study**

(1) Define a problem statement and rationale of the study based on a thorough literature search
(2) Define subsequent primary and secondary research questions which can potentially be addressed by using primary care data
(3) Enquire a primary care database provider to check institutional eligibility, feasibility of the intended research, and potential costs
(4) Secure funding for data acquisition (non-industry funding, if possible)
(5) Register the study in a study database (such as https://clinicaltrials.gov) and publish a study protocol, including a detailed statistical analysis plan, on an open science platform (such as https://osf.io)
(6) Apply for data from the primary care database provider
(7) Acquire the data and store them in compliance with the database provider’s access licence
(8) Conduct the analyses according to the a priori study protocol
(9) Publish a scientific report taking into account relevant reporting guidelines (such as the REporting of studies Conducted using Observational Routinely-collected Data (RECORD) guideline(Benchimol et al., 2015)), including a description of any deviations from the original study protocol, and the statistical code
(10) Delete the database’s original patient datafiles in compliance with the access licence

For academic institutions, the organisation-wide licence to access all of THIN data for unlimited number of studies for a non-commercial purpose costs from €70,000 + tax per annum; whereas project specific data-cuts are priced by patient cohort size, costing €17,000 to €53,000. THIN can also administer survey questionnaires to a sub-group of participating practices, and the costs are approximately €27,000 with completion rates usually above 90%.

The QResearch website contains information for academic researchers wishing to apply for access to the database: https://www.qresearch.org/information. An Advisory Board draws up criteria for access that are applied by a Scientific Committee. Most importantly, data are only released to academics employed by UK universities, and at least one member of the research team must be a medically qualified academic registered with the General Medical Council who signs the guarantee. The cost of a data extraction will be estimated, taking account of the complexity of the data required and the time taken to extract it.

## 5 Strengths and weaknesses of using routine primary care databases

Primary care databases have been mainly used for observational research such as cross-sectional studies, case control studies, and cohort studies (including large pharmacovigilance and pharmaco-epidemiologic studies (Ghosh et al., 2019, Herrett et al., 2015). Example case studies are briefly described in Box 2, Box 3, Box 4, Box 5, Box 6.

Box 2 **Case study on smoking cessation treatment and the risk of depression,suicide and self-harm in the Clinical Practice Research Datalink (CPRD)**

Thomas et al. investigated the neuropsychiatric safety of the currently UK licensed smoking cessation treatments varenicline, bupropion, and nicotine replacement therapy (NRT) using CPRD data (Thomas et al., 2013). At the time of the study, there were serious concerns from spontaneous reporting systems regarding an elevated risk of suicides and suicidal ideation/behaviour associated with the use of varenicline. This had resulted in the US Food and Drug Administration placing its most severe safety warning - a Black Box warning - on varenicline’s product labelling from 2009, three years after varenicline was first licensed. In Europe, varenicline carried a Black Triangle warning. As suicide is a rare event, randomised controlled trials and *meta*-analyses of trials would be unlikely to have a large enough sample size to have enough statistical power to detect this adverse event. Therefore, the authors used data from the CPRD to carry out a large observational study.

Observational pharmacoepidemiological studies are prone to certain limitations such as confounding by indication. This may arise if study participants who receive a particular treatment are systematically different from those participants who receive a comparator treatment. For example, they may be sicker with more co-morbidities. The authors used conventional and novel methods (multivariable regression, propensity score methods, and instrumental variable analyses) to address the issue of confounding by indication. More details on the methods can be obtained from the paper (Thomas et al., 2013).

There was no evidence that patients prescribed either varenicline or bupropion had higher risks of fatal or non-fatal self-harm or treated depression compared with those received NRT using all methods.

Box 3 **Case study on the risk of death during and after opiate substitution treatmentin primary care: prospective observational study in the UK Clinical Practice Research Database (CPRD)**

Cornish et al. investigated the effect of opiate substitution treatment (OST) at the beginning and end of treatment and according to duration of treatment using the General Practice Research Database (GPRD), a precursor to the CPRD (Cornish, Macleod, Strang, Vickerman, & Hickman, 2010). The study examined mortality rates and rate ratios comparing periods in and out of treatment which was adjusted for a range of confounding factors such as age, sex, calendar year, and comorbidity using Poisson regression. They also examined standardised mortality ratios comparing the mortality with general population mortality rates. The authors found a 3.1 times increased mortality rate at the start of OST compared to the rate during the rest of the treatment period and a 9 times increased risk of mortality in the first two weeks immediately after stopping treatment compared with the baseline risk of mortality during treatment.

Box 4 **Case study on the impact of buprenorphine and methadone on mortality: aprimary care cohort study in the UK Clinical Practice Research Database (CPRD)**

Hickman et al. investigated whether opiate substitution treatment (OST) with buprenorphine or methadone was associated with a larger reduction in all-cause mortality (ACM) and mortality from opioid drug related poisoning (DRP).(Hickman et al., 2018) They carried out a cohort study and used linkage data between clinical records from the CPRD and the UK’s Office for National Statistics (ONS) mortality database. They adjusted for confounding factors using propensity score methods. OST with buprenorphine was found to be associated with a lower risk of ACM and DRP compared with methadone.

Box 5 **Case study on evaluating the impact offinancial incentives on delivery of screening and brief advice for alcohol in English primary care using The Health Improvement Network (THIN) database**

O’Donnell et al used THIN data to evaluate the impact of the introduction and withdrawal of financial incentives on the delivery of alcohol screening and brief advice in English primary care (Amy O'Donnell et al., 2020). Records were extracted for all newly registered patients aged 16 and over. Lists of Read codes were devised to capture any clinical or administrative activity corresponding to the three outcome indicators of interest, the rates (percentages) of patients who had been: (1) screened for higher-risk drinking using a validated screening questionnaire (Bush et al., 1998, Hodgson et al., 2002, O'Donnell and Kaner, 2013, Saunders et al., 1993) or questions to ascertain their level of alcohol consumption; (2) screened *and* identified as higher-risk drinkers based on UK guidelines (NICE, 2010); and (3) screened, identified as higher-risk drinkers *and* received brief advice about their drinking. Interrupted time series analysis (ITS) techniques were used to analyse the Read code data (Penfold and Zhang, 2013, Wagner et al., 2002). Monthly rates (%) and 95% confidence intervals for each outcome indicator were calculated for the period 1st January 2006 to 31st December 2016. Rates were plotted graphically to allow visualisation of trends over time, including an initial assessment of any change occurring pre- and post- the two intervention points of interest: (1) introduction of the alcohol DES on 1st April 2008; (2) withdrawal of financial incentives on 31st March 2015. Segmented regression analysis was then used to quantify the magnitude of the impact of the two interventions on our outcomes of interest.

Box 6 **Case study on the prevalence andtreatment of opioid use disorder with buprenorphine across six healthcare systems in the United States**

Lapham et al. reported on the prevalence and treatment rates of opioid use disorder in primary care within six different systems (Lapham et al., 2020). While treatment of this disorder with buprenorphine is intended to be provided in the context of primary care, little is known or empirically documented on the prevalence within primary care or whether patients are able to receive this critical medication for their opioid use disorder. Any adult with two or more visits in any of the six systems between October 2013 and September 2016 were included in the master dataset for evaluation; this included, among several other measures, all International Statistical Classification of Diseases and Related Health Problems (ICD)-9 and ICD-10 codes indicative of an opioid use disorder and whether the patient receive buprenorphine. Out of more than 1.3 million patients, about 14,000 (roughly 1%) has a documented opioid use disorder (adjusted for age, race, ethnicity, gender and health system). Among this group of patients, only 21% received buprenorphine. Some sub-groups had significantly worse access to buprenorphine, including: older individuals, those with great comorbidity burden, women, Black/African American, Hispanic (compared to white), those without commercial insurance, non-cancer pain and a mental health disorder. This group also had proportionally more emergency department visits and hospitalizations. Only one in five patients with opioid use disorder received buprenorphine among six large health systems representing 1.3 million patients. Unfortunately, the reported disparities in access to buprenorphine have become all-too common in the published literature, but are nevertheless discouraging as these clinically disadvantaged sub-groups bear the brunt of the ongoing opioid epidemic. This work does, however, highlight potential opportunities at the system-level, which is something that many other designs would not be capable of uncovering.

### 5.1 Strengths

The use of routine primary care databases for research has several strengths. First, research findings have high external validity. In a country where almost everyone is registered at a general practice, patient data collected from hundreds of practices across the country can be regarded as representative of the population in terms of sociodemographic characteristics (Herrett et al., 2015). Contrary to randomised controlled trials, which are usually conducted in a highly selective group of patients, findings based on primary care database research can thus be readily applied to the ‘real world’ patient population. Furthermore, it allows for the use of study designs where patients with a particular disorder or treatment can be compared with control patients who do not have that disease or are on a different treatment.

Second, data analyses can have high statistical power. Merging information from hundreds of general practices yields a dataset with millions of patients, which is far beyond the sample size of any randomised controlled trial (and even *meta*-analysis) or original prospective cohort study. This allows estimations with a high level of statistical precision and the analysis of rare exposures and outcomes (Herrett et al., 2015). For example, a database study looking at the safety of pharmacotherapy for smoking cessation was able to include 164,766 patients to investigate the association with rare serious adverse events such as cerebral infarction (incidence rates between 6 and 17 events per 1,000 patients per year, depending on the type of pharmacotherapy) and self-harm (5–10 events per 1,000 patients per year) (Kotz et al., 2015).

Third, long-term longitudinal data are available. Many people are registered with the same GP for years and have multiple contacts (and therefore database entries) with the practice. A query of the CPRD, for example, showed that in 2015 active patients (i.e., those alive and registered at that time) had a median follow-up duration of 9.4 years (interquartile range 3.4–13.9 years) (Herrett et al., 2015). This enables research into interventions with long-term outcomes and diseases with long latency and would also point to novel points of prevention once a thorough understanding of a disease’s aetiology is understood (Herrett et al., 2015).

Fourth, primary care data can be linked to other data. As mentioned earlier, some primary care database can be linked to data sources such as official death registrations, hospitalisations, mental health, and cancer registrations (Padmanabhan et al., 2019). Data linkage can improve the completeness of patient information for research purposes and the validity of research studies (Padmanabhan et al., 2019).

Finally, a major opportunity lies in ready access of ‘real-world’ patient data for research without the need to spend massive costs and years of time for data collection, meaning studies can be conducted ad hoc and in real time, if necessary. For example, the COVID-19 pandemic started to affect Europe in spring 2020, and several cohort studies on the risks of disease progression using primary care data from >8 million people were already published in autumn of the same year (Clift et al., 2020, Hippisley-Cox et al., 2020).

### 5.2 Weaknesses

The use of primary care databases for research also has several weaknesses, including some standard limitations of using routinely collected medical data, described more fully to follow (Herrett et al., 2015). With regard to addiction research specifically, the quality and accessibility of primary substance use data will also be shaped by the extent to databases are integrated within the wider health system information infrastructure. In some countries, like the US for example, addiction treatment varies from region to region or state to state and is often fragmented. This can lead to disjointed and inconsistent care (Barry and Huskamp, 2011, Boudreau et al., 2020, Dennis and Scott, 2007, Lapham et al., 2020). While the data captured in one region or state may be well-documented and easy to access, the same may be true for another state or region, but the two systems of data capture are not necessarily easy to harmonize (Boudreau et al., 2020, Lapham et al., 2020).

Another potential disadvantage is the complexity of primary care data, which requires a thorough understanding of the database structure and specific knowledge, skills, and software solutions for processing large datasets and enabling statistical analyses. This can be overcome if the research team includes a strong data scientist or bioinformaticist with expertise in data linkage, manipulation and re-labelling of variables in large, batch form, using state-of-the-art analytic tools. There are also numerous weaknesses associated with the use of observational study designs in general that also apply to research using primary care databases. A recent scoping review of 117 pharmaco-epidemiologic studies that used secondary health databases found key sources of bias, including confounding by indication, residual or unmeasured confounding; outcome misclassification; and immortal time bias (i.e., a follow-up period of a cohort in which death cannot occur because of exposure definition) (Prada-Ramallal, Takkouche, & Figueiras, 2019).

Bias and confounding can be dealt with at the design stage of a study or at the analysis stage (Strom, Kimmel, & Hennessy, 2019). The lack of available data on potential confounding factors is one of the main problems in observational studies utilising large primary care databases (Prada-Ramallal et al., 2019). If data are available on potential confounding factors, careful selection is important (VanderWeele, 2019), and methods can be used such as matching, restriction, or new user designs in the design stage. Stratification, multivariable regression techniques, and propensity score techniques may be used in the analysis stage where confounding factors have been measured. When confounding factors have not been measured, other methods such as sensitivity analyses and more novel methods such as instrumental variable analyses or Bayesian network analyses can be used (Rassen, Brookhart, Glynn, Mittleman, & Schneeweiss, 2009). In many cases the amount of data on a given patient is high which can help to reduce this bias.

### 5.3 Considerations for the statistical analysis of primary care data

When configuring the study design and statistical analysis plan, it is important to recognise that these clinical data were not collected with the primary intent of being used for research purposes. This introduces a variety of considerations that must be accounted for including the impact of a variety of possible confounders on any observed associations, and the impact of site- and system-level sources of variance.

Another important consideration is to understand the purpose of any analytic plan before embarking on the analysis. When examining primary care databases for the purpose of addiction-related research questions, some questions may simply be about characterizing the data elements available across sites and systems. Often, such analyses are somewhat simple and descriptive techniques such as calculating measures of central tendency and spread (Boudreau et al., 2020, Lapham et al., 2020). Here, the primary research question(s) might focus on whether there is a difference in rates of addiction across sites or symptoms in a cross-sectional analysis. A subsequent question may examine longitudinal measures of association; for example, whether access to treatment is a predictor of whether one receives effective care for a diagnosed opioid use disorder. Another, more complex analysis that intends to demonstrate causal inference may make use of instrumental variables. This has become a more commonly used method of analysis in recent years in light of the criticisms levelled against clinical trials around their ability to demonstrate true effects and to subsequently be replicated. However, use of such analyses for the purpose of demonstrating cause and effect has been demonstrated to be difficult even under ideal circumstances. We therefore would recommend much caution when attempting to execute analyses of this type.

One critical step when preparing the data for analysis is harmonization of data elements across sites or systems. As when undertaking a *meta*-analysis, this is one of the most critical steps that all subsequent analyses will rely on. This is not based on advanced statistical techniques, but one of sound and defensible decision making amongst a competent and experienced team that is prepared to defend their decisions to a larger audience of reviewers. Having the requisite expertise, such as a skilled data scientist or bioinformaticist, will help operationalize the clinical decisions made, as noted above. It is important to note here that no analytic plan can make up for taking advantage of optimal design choices early on in the data preparation phases.

Taking this a step further is the importance of writing and pre-registering a detailed statistical analysis plan using available repositories such as the Open Science Framework (https://osf.io), prior to conducting the analysis (Tackett, Brandes, & Reardon, 2019). As with other types of research, increasing attention is paid to this important issue in order to add to the robustness and trustworthiness of such analyses. Indeed, it is often a pre-requisite for randomised clinical trials to pre-register their analytic plan before submission for peer-review. A related consideration is the importance of using reporting guidelines when planning out both the analysis and writing up the method and results once the study is completed. A guideline of particular importance is RECORD (The REporting of Studies Conducted Using Observational Routinely-Collected Health Data) (Benchimol et al., 2015), which builds on the STROBE (Strengthening the Reporting of Observational Studies in Epidemiology) (von Elm et al., 2008) guideline and provides more operationalised description of the necessary data elements and reporting procedures necessary.

Finally, one important statistical consideration we have found useful when conducting analyses designed to show at least an association if not cause and effect is the use of sensitivity analyses. Such analyses can serve an important function when trying to address a concern about whether to include certain variables. Given the complexity of analyses with this kind of data, sensitivity analyses serve an important ‘stress test’ function to examine whether the associations in question remain robust.

### 5.4 Additional considerations to inform the design and interpretation of research

Electronic patient records possess a number of key advantages as an information source for researchers. For example, as such data are by definition collected as part of the routine management and delivery of healthcare services, they represent a cost-effective and relatively unobtrusive means of gathering information (McKee, 1993). This is particularly the case when compared with direct observation or the introduction of behavioural measures, both of which are complex and costly to use (Hrisos et al., 2009) and introduce the possibility of the ‘Hawthorne Effect’, whereby the act of participating in research can influence clinical practice (Roethlisberger & Dickson, 1939).Furthermore, routine health data offer an especially comprehensive information source: they are available in multiple settings and provide a rich source of information about large numbers of patients (Powell, Davies, & Thomson, 2003), in many cases, providing details of a patient’s diagnoses, management and health outcomes over the full life course (Gnani & Majeed, 2006).

At the same time, there are also several acknowledged difficulties of using routine primary care data for research purposes (de Lusignan and van Weel, 2006, Gray et al., 2003, Terry et al., 2010). This is unsurprising, given that the different needs and priorities of clinical users, as opposed to the research users, wherein it will inform the degree of care or consistency with which such data is recorded in day-to-day practice (Weiskopf & Weng, 2013). When considering whether primary care data are of sufficient quality for research purposes, Weiskopf and Weng suggest we consider three core dimensions: completeness, correctness (or accuracy), and currency (or timeliness) (Powell et al., 2003, Weiskopf and Weng, 2013).

Looking first at the question of whether primary care data can be considered *complete.* This dimension is closely related to specificity; i.e. to what extent can we assume that every ‘real world’ instance of a concept (diagnosis, treatment, characteristic, etc.) has been recorded in patient records? (W. & Wagner, 1997) For ‘complete’ data on the diagnosis of an alcohol use disorder, for example, this would mean that every patient known to have an alcohol use disorder in a given population (e.g., practice registered list) would have that fact recorded in their electronic record. In ‘real world’ practice, however, ‘completeness’ can depend on the type of clinical behaviour or action being recorded (with more thorough records for actions relating to physical examination, laboratory tests, and screening services compared to counselling services or lifestyle advice (Hrisos et al., 2009)), and of course whether patients themselves actively present for screening, assessment or treatment (Kane, Wellings, Free, & Goodrich, 2000). For example, individuals who are without access to basic care routinely in the US will often receive their primary care through urgent and even emergency room care. This same problem extends to access to appropriate care for substance use disorders, where the lack of complete data may represent a larger social injustice at work (King, Englander, Priest, Korthuis, & McPherson, 2020). Additionally, the continued stigma attached to alcohol and other substance use may affect both patients’ willingness to disclose their substance use status during consultations, and primary care clinicians’ readiness to record that information (McNeely et al., 2018).

Second, there is the question of data *correctness* or accuracy. For some, this dimension is analogous with the measure of positive predictive value (the proportion of positive data that are true positives (Hassey, Gerrett, & Wilson, 2001)). However, correctness relates not just to the question of whether we can say that the information contained in routine medical records is ‘true’ (and thus in part linked to completeness), but also to whether the data itself has been recorded correctly. In this respect, it is important to be aware that information recorded on GP systems is seldom homogeneous (Waize Tai, Anandarajah, Dhoul, & De Lusignan, 2007). Primary care datasets are a collective effort, comprised of input from multiple individuals, who have varied professional roles and responsibilities, and work in different practice contexts (Department of Health et al., 2011). Such factors can combine to compromise the reliability of routine data, further compounded by the fact that several individuals may be involved in data collection and recording over time (Strange, Zyzanski, Fedirko Smith, & al., 1998). Additionally, as already highlighted, under the Read code system, a given piece of information may be recorded in several different ways. It may be coded or written in free text, which may contain acronyms or abbreviations. Coding may be based on using national, local or even practice level recording guidelines and/or codes themselves. Although some local Read codes are created by suppliers and are essential to support normal system functions, others have been developed to augment or in some cases duplicate existing Read codes. Such codes cannot be rendered fully interoperable (i.e. cannot be understood if transferred to other supplier systems) (Crosson et al., 2009), and thus undermine the consistency of patient health records. Full rollout of SNOMED CT in the UK should help address some of these historical inconsistences. However, there is also a need for appropriate professional bodies to develop and actively implement minimum recording standards for alcohol, tobacco, and other substances in clinical settings, as per the recent initiative from the UK Royal College of Physicians (Haroon et al., 2018, Royal College of Physicians, 2021a, Royal College of Physicians, 2021b).

Third, data quality is also affected by its *currency* or timeliness. For example, whether there is a time-lag between the capture and the publication or availability of routine data (Kane et al., 2000). A key advantage of primary care data is generally considered to be its immediacy in comparison to other routine data sets. However, and linked to the above issues of ensuring accurate and homogenous coding practices, there is possibly more doubt over the extent to which such data are actually *available,* whether that concerns accessibility from a researcher’s perspective, or that of the practitioner themselves. In general, structured data (e.g., coded information) will be more rapidly available than free text; however, as already highlighted, inconsistent coding and the use of practice-based euphemisms may reduce accessibility. Further, the architecture of the computerised practice record also impacts on the ease with which information can be accessed. Not all systems facilitate effective data linkage and in particular, the lack of a reliable unique identifier for each patient makes linkage with other systems challenging (de Lusignan & van Weel, 2006).

Underlying these three standard dimensions of data quality are the additional issues of *relevancy* and *purpose*. For example, primary care clinicians and administrators are most likely to record information if they believe it to be important or relevant to a given situation or context at the time of recording. In this respect, there is evidence that pay for performance schemes can distort coding practice, with clinicians tending to prioritise recording of data corresponding to delivery of incentivised areas of care (Prytherch, Briggs, Weaver, Schmidt, & Smith, 2005). Additionally, as digitalised health records provide an ever-more accessible resource for researchers, there are also some ethical concerns that should be acknowledged. For some, using private medical data for purposes other than the immediate health needs of the individual patient potentially represents a breach of confidentiality (Kane et al., 2000), leading Van Der Lei to argue that electronic data should *‘be used only for the purposes for which they were collected’* (van der Lei, 1991). Whether such a breach of confidentiality is justified, is a subject for continued debate, and as Foster and Young highlight, often rests somewhat uncomfortably on conventional and morally simplistic assumptions of research as a process which implicitly ‘benefits’ the public ‘other’ (Foster & Young, 2012). However, not all research is ‘good’ research (objective, independent, beneficent), and the use (or misuse) of routine health data can result in some real and damaging consequences for patients that extend well beyond their initial interaction with the health system. For example, allowing insurance companies access to certain types of medical data could seriously jeopardise a patient’s financial status, affecting their ability to access credit or to secure health insurance (Cayton & Denegri, 2003).

## 6 Conclusion

Primary care databases extract and combine on a regular basis routine data from electronic patient records. Researchers can get access to such databases to address questions in addiction research. The strengths of using primary care databases for research include high external validity of research findings, high statistical power, the availability of long-term longitudinal data, linkage to other healthcare databases, and the possibility to conduct research at relatively low cost and within a short period of time. However, researchers need to be aware that primary care data are limited with regard to completeness, accuracy, scope, and standardisation of definitions for identifying exposures, outcomes and diagnoses. Furthermore, observational designs using routine care data are prone to various forms of bias and confounding. Despite these limitations, primary care databases remain a valuable source for innovative addiction research.

## Funding

Sterling McPherson’s work on this manuscript was supported in part by a grant from the National Institute on Drug Abuse, USA (UG1 DA013714). Kyla Thomas is currently funded by a 10.13039/501100000272National Institute for Health Research (NIHR) UK postdoctoral fellowship (grant reference: PDF‐2017‐10‐068). Amy O'Donnell is funded by a NIHR Advanced Fellowship (grant reference: NIHR300616). The views expressed are those of the authors and not necessarily those of the National Health Service, the NIHR, or the Department of Health and Social Care.

### CRediT authorship contribution statement

**Daniel Kotz:** Conceptualization. **Amy O'Donnell:** Conceptualization. **Sterling McPherson:** Conceptualization. **Kyla H. Thomas:** Conceptualization.

## Declaration of Competing Interest

The authors declare that they have no known competing financial interests or personal relationships that could have appeared to influence the work reported in this paper.

## References

[b0005] Barry C.L., Huskamp H.A. (2011). Moving beyond parity–mental health and addiction care under the ACA. New England Journal of Medicine.

[b0010] Benchimol E.I., Smeeth L., Guttmann A., Harron K., Moher D., Petersen I., Committee R.W. (2015). The REporting of studies Conducted using Observational Routinely-collected health Data (RECORD) Statement. PLoS Medicine.

[b0015] Blak B.T., Thompson M., Dattani H., Bourke A. (2011). Generalisability of The Health Improvement Network (THIN) database: Demographics, chronic disease prevalence and mortality rates. Informatics Primary Care.

[b0020] Boudreau D.M., Lapham G., Johnson E.A., Bobb J.F., Matthews A.G., McCormack J., Bradley K.A. (2020). Documented opioid use disorder and its treatment in primary care patients across six U.S. health systems. Journal of Substance Abuse Treatment.

[b0025] Bush, K., Kivlahan, D. R., McDonell, M. B., Fihn, S. D., Bradley, K. A., & for the Ambulatory Care Quality Improvement, P. (1998). The audit alcohol consumption questions (audit-c): An effective brief screening test for problem drinking. *JAMA Internal Medicine 158*(16), 1789–1795. doi:10.1001/archinte.158.16.1789. 10.1001/archinte.158.16.1789 9738608

[b0030] Cayton H., Denegri S. (2003). Is what's mine my own?. Journal of Health Service Research and Policy.

[b0035] Clift A.K., Coupland C.A.C., Keogh R.H., Hemingway H., Hippisley-Cox J. (2020). COVID-19 Mortality Risk in Down Syndrome: Results From a Cohort Study Of 8 Million Adults. Annals of Internal Medicine.

[b0040] Cornish R., Macleod J., Strang J., Vickerman P., Hickman M. (2010). Risk of death during and after opiate substitution treatment in primary care: Prospective observational study in UK General Practice Research Database. BMJ.

[b0045] Crosson J., Ohman-Strickland P., Campbell S., Phillips R., Roland M., Kontopantlis E., Crabtree B. (2009). A comparison of chronic illness care quality in US and UK family medicine practices prior to pay-for-performance performance initiatives. Family Practice.

[b0050] de Lusignan S., van Weel C. (2006). The use of routinely collected computer data for research in primary care: Opportunities and challenges. Family Practice.

[b0055] Dennis M., Scott C.K. (2007). Managing addiction as a chronic condition. Addiction Science & Clinical Practice.

[b0060] Department of Health, Royal College of General Practitioners, & British Medical Association. (2011). *The Good Practice Guidelines for GP electronic patient records v4*. Retrieved from London.

[b0065] Foster V., Young A. (2012). The use of routinely collected patient data for research: A critical review. Health.

[b0070] Ghosh R.E., Crellin E., Beatty S., Donegan K., Myles P., Williams R. (2019). How Clinical Practice Research Datalink data are used to support pharmacovigilance. Therapeutic advances in drug safety.

[b0075] Gnani, S., & Majeed, A. (2006). *A user's guide to data collected in primary care in England*. Retrieved from London.

[b0080] Gray J., Orr D., Majeed A. (2003). Use of Read codes in diabetes management in a south London primary care group: Implications for establishing disease registers. British Medical Journal.

[b0085] Gregory, S. (2009). *General practice in England: An overview*. Retrieved from London.

[b0090] Haroon S., Wooldridge D., Hoogewerf J., Nirantharakumar K., Williams J., Martino L., Bhala N. (2018). Information standards for recording alcohol use in electronic health records: Findings from a national consultation. BMC Medical Informatics and Decision Making.

[b0095] Hassey A., Gerrett D., Wilson A. (2001). Information in practice: A survey of validity and utility of electronic patient records in a general practice. BMJ.

[b0100] Herrett E., Gallagher A.M., Bhaskaran K., Forbes H., Mathur R., van Staa T., Smeeth L. (2015). Data Resource Profile: Clinical Practice Research Datalink (CPRD). International Journal of Epidemiology.

[b0105] Hickman M., Steer C., Tilling K., Lim A.G., Marsden J., Millar T., Macleod J. (2018). The impact of buprenorphine and methadone on mortality: A primary care cohort study in the United Kingdom. Addiction.

[b0110] Hippisley-Cox J., Tan P.S., Coupland C. (2020). Risk of severe COVID-19 disease with ACE inhibitors and angiotensin receptor blockers: Cohort study including 8.3 million people. Heart.

[b0115] Hodgson R.J., Alwyn T., John B., Thom B., Smith A. (2002). The FAST Alcohol Screening Test. Alcohol & Alcoholism.

[b0120] Hrisos S., Eccles M., Francis J., Dickinson H., Kaner E., Beyer F., Johnston M. (2009). Are there valid proxy measures of clinical behaviour? A systematic review. Implementation Science.

[b0125] Kane R., Wellings K., Free C., Goodrich J. (2000). Uses of routine data sets in the evaluation of health promotion interventions: Opportunities and limitations. Health Education.

[b0130] King C., Englander H., Priest K.C., Korthuis P.T., McPherson S. (2020). Addressing Missing Data in Substance Use Research: A Review and Data Justice-based Approach. Journal of Addiction Medicine.

[b0135] Kotz D., Viechtbauer W., Simpson C., van Schayck O.C., West R., Sheikh A. (2015). Cardiovascular and neuropsychiatric risks of varenicline: A retrospective cohort study. The Lancet Respiratory Medicine.

[b0140] Lapham G., Boudreau D.M., Johnson E.A., Bobb J.F., Matthews A.G., McCormack J., Bradley K.A. (2020). Prevalence and treatment of opioid use disorders among primary care patients in six health systems. Drug and Alcohol Dependence.

[b0145] Lawrenson R., Williams T., Farmer R. (1999). Clinical information for research; the use of general practice databases. Journal of Public Health Medicine.

[b0150] McKee M. (1993). Routine data: A resource for clinical audit?. Quality in Health Care.

[b0155] McNeely J., Kumar P.C., Rieckmann T., Sedlander E., Farkas S., Chollak C., Rotrosen J. (2018). Barriers and facilitators affecting the implementation of substance use screening in primary care clinics: A qualitative study of patients, providers, and staff. Addiction Science & Clinical Practice.

[b0160] NHS Digital. (2016). *Guidance for Primary Care: Transitioning from Read to SNOMED CT: Version 1*. Retrieved from London.

[b0165] NICE. (2010). *Alcohol-use disorders: preventing the development of hazardous and harmful drinking: NICE public health guidance 24*. Retrieved from London.

[b0170] O'Donnell A., Angus C., Hanratty B., Hamilton F.L., Petersen I., Kaner E. (2020). Impact of the introduction and withdrawal of financial incentives on the delivery of alcohol screening and brief advice in English primary health care: An interrupted time–series analysis. Addiction.

[b0175] O'Donnell A., Kaner A., Boyle B., Zatonski Lowenfels, Brawley Burns, Rehm (2013). Alcohol, Science, Policy and Public Health.

[b0180] Padmanabhan S., Carty L., Cameron E., Ghosh R.E., Williams R., Strongman H. (2019). Approach to record linkage of primary care data from Clinical Practice Research Datalink to other health-related patient data: Overview and implications. European Journal of Epidemiology.

[b0185] Penfold R.B., Zhang F. (2013). Use of interrupted time series analysis in evaluating health care quality improvements. Academic Pediatric.

[b0190] Powell A., Davies H., Thomson R. (2003). Using routine comparative data to assess the quality of health care: Understanding and avoiding common pitfalls. Quality and Safety in Health Care.

[b0195] Prada-Ramallal G., Takkouche B., Figueiras A. (2019). Bias in pharmacoepidemiologic studies using secondary health care databases: a scoping review. BMC Medical Research Methodology.

[b0200] Prytherch D., Briggs J., Weaver P., Schmidt P., Smith G. (2005). Measuring clinical performance using routinely collected clinical data. Medical Informatics and the Internet in Medicine.

[b0205] Rassen J.A., Brookhart M.A., Glynn R.J., Mittleman M.A., Schneeweiss S. (2009). Instrumental variables I: Instrumental variables exploit natural variation in nonexperimental data to estimate causal relationships. Journal of Clinical Epidemiology.

[b0210] Robinson D., Schulz E., Brown P., Price C. (1997). Updating the Read Codes: User-interactive Maintenance of a Dynamic Clinical Vocabulary. Journal of the American Medical Informatics Association.

[b0215] Roethlisberger F., Dickson W. (1939).

[b0220] Royal College of Physicians. (2021a). Information standards for recording alcohol use in electronic health records [accessed 14 December 2021 at: https://www.rcplondon.ac.uk/projects/outputs/information-standards-recording-alcohol-use-electronic-health-records].

[b0225] Royal College of Physicians. (2021b). Information standards for recording tobacco use in electronic health records [accessed 14 December 2021 at: https://www.rcplondon.ac.uk/projects/outputs/information-standards-recording-tobacco-use-electronic-health-records].

[b0230] Saunders J.B., Aasland O.G., Babor T.F., de la Fuente J.R., Grant M. (1993). Development of the Alcohol Use Disorders Identification Test (AUDIT): WHO Collaborative Project on Early Detection of Persons with Harmful Alcohol Consumption II. Addiction.

[b0235] SNOMED International. (2020). SNOMED-CT: Five step briefing. Strange, K., Zyzanski, S., Fedirko Smith, T., & al., e. (1998). How valid are medical records and patient questionnaires for physician profiling and health services research? A comparison with direct observation of patient visits. *Medical Care, 38,* pp. 851–867. 10.1097/00005650-199806000-00009 9630127

[b0240] Strange K., Zyzanski S., Fedirko Smith T. (1998). How valid are medical records and patient questionnaires for physician profiling and health services research? A comparison with direct observation of patient visits. Medical Care.

[b0245] Strom B.L., Kimmel S.E., Hennessy S. (2019).

[b0250] Tackett J.L., Brandes C.M., Reardon K.W. (2019). Leveraging the Open Science Framework in clinical psychological assessment research. Psychological Assessment.

[b0255] Terry A.L., Chevendra V., Thind A., Stewart M., Marshall J.N., Cejic S. (2010). Using your electronic medical record for research: A primer for avoiding pitfalls. Family Practice.

[b0260] THIN (The Health Improvement Network). (2020). THIN: The Health Improvement Network.

[b0265] Thomas K.H., Martin R.M., Davies N.M., Metcalfe C., Windmeijer F., Gunnell D. (2013). Smoking cessation treatment and risk of depression, suicide, and self harm in the Clinical Practice Research Datalink: Prospective cohort study. BMJ.

[b0270] Townsend P., Phillimore P., Beattie A. (1988).

[b0275] UK Terminology Centre. (2011). *Why migrate to SNOMED CT?* . Retrieved from London.

[b0280] van der Lei J. (1991). Use and abuse of computer-stored medical records. Methods of Information in Medicine.

[b0285] VanderWeele T.J. (2019). Principles of confounder selection. European Journal of Epidemiology.

[b0290] Vezyridis P., Timmons S. (2016). Evolution of primary care databases in UK: A scientometric analysis of research output. BMJ Open.

[b0295] von Elm E., Altman D.G., Egger M., Pocock S.J., Gotzsche P.C., Vandenbroucke J.P. (2008). The Strengthening the Reporting of Observational Studies in Epidemiology (STROBE) statement: Guidelines for reporting observational studies. Journal of Clinical Epidemiology.

[b0300] Hogan W.R., Wagner M. (1997). Accuracy of data in computer-based patient records. Journal of the American Medical Informatics Association.

[b0305] Wagner A.K., Soumerai S.B., Zhang F., Ross-Degnan D. (2002). Segmented regression analysis of interrupted time series studies in medication use research. Journal of Clinical Pharmacy and Therapeutics.

[b0310] Waize Tai T., Anandarajah S., Dhoul N., De Lusignan S. (2007). Variation in clinical coding lists in UK general practice: A barrier to consistent data entry?. Informatics in Primary Care.

[b0315] Weiskopf N.G., Weng C. (2013). Methods and dimensions of electronic health record data quality assessment: Enabling reuse for clinical research. Journal of the American Medical Informatics Association.

[b0320] Williams T., van Staa T., Puri S., Eaton S. (2012). Recent advances in the utility and use of the General Practice Research Database as an example of a UK Primary Care Data resource. Therapeutic Advances in Drug Safety.

[b0325] Wolf A., Dedman D., Campbell J., Booth H., Lunn D., Chapman J., Myles P. (2019). Data resource profile: Clinical Practice Research Datalink (CPRD) Aurum. International Journal of Epidemiology.

